# Nanobody-mediated SPECT/CT imaging reveals the spatiotemporal expression of programmed death-ligand 1 in response to a CD8^+^ T cell and iNKT cell activating mRNA vaccine

**DOI:** 10.7150/thno.85106

**Published:** 2023-10-09

**Authors:** Thomas Ertveldt, Sofie Meulewaeter, Yannick De Vlaeminck, Oscar Olarte, Katrijn Broos, Serge Van Calenbergh, Stephanie Bourgeois, Joke Deprez, Yves Heremans, Cleo Goyvaerts, Willem Staels, Stefaan De Smedt, Heleen Dewitte, Nick Devoogdt, Marleen Keyaerts, Rein Verbeke, Kurt Barbé, Ine Lentacker, Karine Breckpot

**Affiliations:** 1 Laboratory for Molecular and Cellular Therapy, Vrije Universiteit Brussel, Laarbeeklaan 103, B-1090 Brussels, Belgium.; 2 Ghent research Group on Nanomedicines, Laboratory of Physical Pharmacy and General Biochemistry, Department of Pharmaceutics, Ghent University, Ottergemsesteenweg 460, B-9000 Gent, Belgium.; 3 Cancer Research Institute Ghent (CRIG), Ghent University Hospital, Ghent University, Ghent B-9000, Belgium.; 4 Biostatistics and Medical Informatics Research Group, Vrije Universiteit Brussel, Laarbeeklaan 103, B-1090 Brussels, Belgium.; 5 Laboratory of Medicinal Chemistry, Department of Pharmaceutics, Ghent University, Ottergemsesteenweg 460, B-9000, Belgium.; 6 Beta Cell Neogenesis (BENE), Vrije Universiteit Brussel, Laarbeeklaan 103, Brussels, Belgium.; 7 Visual and Spatial Tissue Analysis (VSTA) Core Facility, Vrije Universiteit Brussel, Laarbeeklaan 103, 1090 Brussels, Belgium; 8 Universitair Ziekenhuis Brussel (UZ Brussel), Department of Pediatrics, Division of Pediatric Endocrinology, Brussels, Belgium.; 9 Medical Imaging department, In Vivo Cellular and Molecular Imaging Laboratory, Vrije Universiteit Brussel, Laarbeeklaan 103, B-1090 Brussels, Belgium.; 10 Nuclear Medicine Department, UZ Brussel, Laarbeeklaan 101, B-1090 Brussels, Belgium.

**Keywords:** melanoma, mRNA vaccine, programmed death-ligand 1, nanobody, single-photon emission computerized tomography/computed tomography

## Abstract

**Rationale:** Although promising responses are obtained in patients treated with immune checkpoint inhibitors targeting programmed death ligand 1 (PD-L1) and its receptor programmed death-1 (PD-1), only a fraction of patients benefits from this immunotherapy. Cancer vaccination may be an effective approach to improve the response to immune checkpoint inhibitors anti-PD-L1/PD-1 therapy. However, there is a lack of research on the dynamics of PD-L1 expression in response to cancer vaccination.

**Methods:** We performed non-invasive whole-body imaging to visualize PD-L1 expression at different timepoints after vaccination of melanoma-bearing mice. Mice bearing ovalbumin (OVA) expressing B16 tumors were i.v. injected with the Galsome mRNA vaccine: OVA encoding mRNA lipoplexes co-encapsulating a low or a high dose of the atypical adjuvant α-galactosylceramide (αGC) to activate invariant natural killer T (iNKT) cells. Serial non-invasive whole-body immune imaging was performed using a technetium-99m (^99m^Tc)-labeled anti-PD-L1 nanobody, single-photon emission computerized tomography (SPECT) and X-ray computed tomography (CT) images were quantified. Additionally, cellular expression of PD-L1 was evaluated with flow cytometry.

**Results:** SPECT/CT-imaging showed a rapid and systemic upregulation of PD-L1 after vaccination. PD-L1 expression could not be correlated to the αGC-dose, although we observed a dose-dependent iNKT cell activation. Dynamics of PD-L1 expression were organ-dependent and most pronounced in lungs and liver, organs to which the vaccine was distributed. PD-L1 expression in lungs increased immediately after vaccination and gradually decreased over time, whereas in liver, vaccination-induced PD-L1 upregulation was short-lived. Flow cytometric analysis of these organs further showed myeloid cells as well as non-immune cells with elevated PD-L1 expression in response to vaccination. SPECT/CT imaging of the tumor demonstrated that the expression of PD-L1 remained stable over time and was overall not affected by vaccination although flow cytometric analysis at the cellular level demonstrated changes in PD-L1 expression in various immune cell populations following vaccination.

**Conclusion:** Repeated non-invasive whole-body imaging using ^99m^Tc-labeled anti-PD-L1 nanobodies allows to document the dynamic nature of PD-L1 expression upon vaccination. Galsome vaccination rapidly induced systemic upregulation of PD-L1 expression with the most pronounced upregulation in lungs and liver while flow cytometry analysis showed upregulation of PD-L1 in the tumor microenvironment. This study shows that imaging using nanobodies may be useful for monitoring vaccine-mediated PD-L1 modulation in patients and could provide a rationale for combination therapy. To the best of our knowledge, this is the first report that visualizes PD-L1 expression upon cancer vaccination.

## Introduction

The treatment of patients with advanced metastatic melanoma has been revolutionized since the development of antibodies that block the inhibitory immune checkpoints programmed death ligand 1 (PD-L1) and its receptor programmed death-1 (PD-1) 1. These antibodies remove the brake that PD-1 enforces on T cell proliferation and anti-tumor activity and show clinical benefit in approximately 40% of the patients 2. Despite this success, up to 60% of patients are not surviving five years after treatment.

Cancer vaccination may be an effective approach to improve the response to anti-PD-L1/PD-1 therapy 3. Several cell-based vaccination studies have been performed focusing primarily on dendritic cells (DCs) as these cells are indispensable for antigen presentation to and activation of melanoma-specific CD8^+^ T cells 4. Furthermore, crosstalk of DCs with T cells is vital for effective anti-PD-1 therapy 5. However, the preparation of these patient-specific DC vaccines is time-consuming, expensive and the outcome of DC vaccination is variable 6,7. Therefore, viral and non-viral strategies to deliver tumor antigens with or without adjuvants to fully activate the T cell stimulatory capacity of DCs *in situ* have been explored 8,9. Of these, mRNA-based vaccines are of major interest as the experience with COVID-19 mRNA-vaccines has proven their worth as a cost-effective, widely adoptable vaccine platform 10.

Galsomes, the mRNA vaccination platform used in this work, consist of cationic lipid-based lipoplexes co-encapsulating tumor antigen encoding mRNA and the glycolipid adjuvant α-galactosylceramide (αGC). After i.v. administration, Galsomes are taken up by DCs which subsequently present tumor antigen-derived peptides in class I major histocompatibility (MHC-I) complexes to CD8^+^ T cells. Furthermore, due to the inclusion of the atypical adjuvant αGC, another T cell with anti-tumor properties, namely invariant natural killer T (iNKT) cells, will get activated as a consequence of the presentation of the glycolipid antigen in the context of CD1d complexes by DCs 11. iNKT cells are innate-like T cells that can exert direct cytotoxic effects on cells upon stimulation of their natural killer activating receptor or by interaction with CD1d-expressing cells. Hence, iNKT cells can kill dedicated tumor cells and modulate the immunosuppressive tumor microenvironment (TME), *e.g.,* by killing alternatively activated immunosuppressive macrophages 12,13. Additionally, activation of iNKT cells results in a rapid burst of interferon-gamma (IFN-γ), which is a pleiotropic cytokine that enhances antigen presentation by DCs and further stimulates activation of CD8^+^ T cells. Furthermore, IFN-γ produced by iNKT cells, but also by CD8^+^ T cells, is indispensable for tumor cell killing 14. Paradoxically, IFN-γ and other cytokines such as IL-6 orchestrate the expression of PD-L1 to dampen immune responses and avoid collateral damage 15,16. As a consequence, PD-L1 can be upregulated in cancer cells, but also in immune cells such as DCs, shortly after immune activation, impairing T cell priming 17,18. We have previously shown that combination therapy of Galsomes with anti-PD-L1 therapy is synergistic and not only improves the therapeutic outcome, but also strengthens immune cell activation upon repeated Galsome vaccination 11. Thus, PD-L1 is not only a barrier that hampers the immune response during the T cell effector phase at the tumor site but also during the T cell activation phase.

Though the opposing antitumor activities of IFN-γ seem contradictory at first glance, this opens avenues to set up effective combination therapies of cancer vaccines with anti-PD-1/PD-L1 therapy, as demonstrated in several clinical trials 19,20. However, most studies combining immune checkpoint blockade with other therapies, operate on a trial-and-error basis and lack the ability to properly gauge PD-L1 expression levels. To optimally set up a treatment regimen based on Galsome vaccination and PD-1/PD-L1 therapy and avoid unneeded administration of immune checkpoint inhibitors in combination with cancer vaccination, it is key to study the onset and dynamics of PD-L1 expression upon Galsome vaccination. This allows to further rationalize and optimize immune checkpoint blockade combination therapy to improve therapeutic outcome whilst minimizing immune-related adverse events (irAE). In this manuscript, we evaluated whole body imaging to provide information on the spatial distribution of PD-L1 expression as well as gain more fundamental insights into the dynamics of PD-L1 expression upon vaccination. Nuclear imaging of PD-L1 using single-photon emission computerized tomography (SPECT) or positron emission tomography (PET) in combination with computed tomography (CT) can be achieved using radiolabeled nanobodies, small yet robust and easy-to-radiolabel antigen binding moieties that are derived from heavy chain-only antibodies that are found in camelids 21-23. Due to its non-invasiveness, this technique allows repeated imaging and can visualize heterogeneity within and between different cancer lesions, in stark contrast to immunohistochemistry of tumor biopsies. We applied a technetium-99m (^99m^Tc)-labeled anti-PD-L1 nanobody 23 to image the dynamic expression of PD-L1 in a mouse melanoma model after Galsome vaccination. Moreover, we assessed the effect of the iNKT cell-ligand αGC on PD-L1 expression by varying the αGC-dose included in the Galsome-vaccine. We generated serial high-resolution images allowing quantification of PD-L1 expression in peripheral organs and tumors. *Ex vivo* analysis using flow cytometry confirmed the results of the quantified images and showed that both non-immune and myeloid cells contributed to PD-L1 expression in the organs that were studied. In conclusion, our data show that PD-L1 imaging could be a useful tool to stratify patients and gauge therapies' eligibility for PD-L1 blockade.

## Material and methods

### Mice

Female C57BL/6 and OT-I mice (6-12 weeks) were purchased from Charles River (France) and handled according to institutional guidelines. Experiments were approved by the ethical committee for use of laboratory animals at Vrije Universiteit Brussel (19-272-1) and Ghent University (20/07).

### Generation and quality control of B16-OVA cells

Murine B16 melanoma cells (ATCC) were lentivirally engineered to express ovalbumin (OVA), resulting in B16-OVA cells. Briefly, the expression cassette of the pLenti-CMV-GFP-Puro transfer plasmid (Addgene), consisting of the cytomegalovirus promotor (CMV) driving the expression of green fluorescent protein (GFP), was replaced with an expression cassette, consisting of the EF1α promotor driving the expression of OVA (pLenti-EF1α-OVA). To that end, the CMV promotor was excised using ClaI-XbaI (Thermo Scientific) and replaced by an EF1α gBlock with matching overhangs (IDT), while GFP was excised using BamHI-SalI (Thermo Scientific) and replaced by an OVA gBlock with matching overhangs (IDT). Second-generation, VSV.G pseudotyped lentiviral particles were produced using pLenti-EF1α-OVA, the packaging plasmid pCMV∆R8.9 and envelope plasmid pMD.G (kind gifts from D. Trono, Geneva) 24. B16 cells were transduced with lentiviral particles at a multiplicity of infection of 10, after which transduced cells were selected using 0.4 mg mL^-1^ puromycin (Sigma-Aldrich) in Dulbecco's modified Eagle's medium (DMEM, Sigma-Aldrich) containing 10% heat-inactivated fetal bovine serum (FBS, Life Technologies), 2 mmol L^-1^ L-glutamine (Sigma-Aldrich), 100 U mL^-1^ penicillin and 0.1 mg mL^-1^ streptomycin (Sigma-Aldrich). Cells were grown at 37°C in a humidified atmosphere with 5% CO_2_. The MycoSEQ mycoplasma detection assay, based on polymerase chain reaction (ThermoFisher Scientific), was used to screen the B16-OVA cell line for mycoplasma contamination.

### CD8^+^ T cell activation assay

B16 cells, B16 cells pulsed with 10 µg mL^-1^ of the OVA-derived peptide SIINFEKL (Anaspec) and B16-OVA cells were co-cultured at a 1:10 ratio with 10^5^ CD8^+^ T cells that were enriched from the spleen of OT-I mice using the CD8α^+^ T cell isolation kit II (Miltenyi Biotec). As controls, CD8^+^ T cells were cultured without further stimulation or with addition of anti-CD3/CD28 antibody coated beads (1/800 dilution) (Gibco). On day 6 of co-culture, supernatants were collected and analyzed via ELISA for IFN-γ production (eBioScience), while cells were analyzed for IFN-γ production by means of flow cytometry.

### mRNA

The genetic code of the fusion protein consisting of the first 80 amino acids of the invariant chain (Ii80) fused to a truncated and non-secreted variant of OVA (tOVA) was cloned into the pGEM-plasmid 25. The pGEM-Ii80tOVA plasmid and pLMCT-fLuc (for synthesis of a thermostable firefly luciferase (fLuc)) were linearized with the restriction enzymes SpeI (Thermo Fisher Scientific) and BfuAI (New England Biolabs, Frankfurt am Main, Germany) respectively and were used as a template for *in vitro* transcription (IVT) of mRNA using the T7 MegaScript kit (Thermo Fisher Scientific) and the Clean CAP AG reagent (TriLink Biotech). The chemically modified nucleotide N1-methylpseudouridine-5'-triphosphate (1-mѰU, Tebu-Bio) was used instead of uridine. The yield, purity, and integrity of the mRNA were analysed as previously described 26.

### Vaccine formulation - Galsomes

Galsomes were prepared by mixing chloroform-dissolved 1,2-dioleoyl-3-trimethylammonium-propane (DOTAP), cholesterol (both from Avanti Polar Lipids), and αGC (synthesized in-house) at a molar ratio of 40:60:0.015 (high αGC-dose) or 40:60:0.0015 (low αGC-dose) in a round-bottom flask. After evaporation of the chloroform under a nitrogen stream, the resulting lipid film was rehydrated in HEPES buffer (20 mM, pH 7.4, Sigma Aldrich) to generate liposomes at a final lipid concentration of 12.5 mM. Liposomes were sonicated in a Branson sonication bath until the dispersion cleared. Complexation with Ii80tOVA mRNA (10 µg) to formulate Galsomes was performed at an NP-ratio of 3 (N referring to the positive charges on the lipids, P referring to the negative charges on the nucleic acids) in an isotonic HEPES buffer containing 5% glucose. In case Galsomes (high dose) were prepared to study biodistribution, 1.2 mol% of the total lipid amount was replaced by the lipophilic dye DiR (Molecular Probes™, Thermo Fisher Scientific) and Galsomes were complexed with fLuc mRNA. Quality controls on the generated Galsomes were done by measuring size, polydispersity, and charge using a Malvern Zetasizer nano-ZS 11.

### Vaccination of melanoma-bearing mice

A total of 3×10^5^ B16-OVA cells were implanted subcutaneously (s.c) into the right thigh of mice that were sedated with 2.5% isoflurane and 1 L.min^-1^ oxygen flow rate (Abbott). Monitoring of tumor growth was performed daily. A caliper was used to measure the dimensions (length and width) of palpable tumors. Tumor volumes were calculated as follows: (width × length^2^)/2. Before treatment, animals were randomized based on tumor volume and were intravenously (i.v.) injected when the tumors reached a size of 166±137 mm^3^ (11 days post inoculation) with a low or a high dose Galsomes containing both 10 µg Ii80tOVA mRNA with 2 or 20 ng αGC, respectively. Mice allocated to the control group received phosphate-buffered saline (PBS, Sigma-Aldrich).

### *In vivo* Galsome distribution

Female C57BL/6 mice (6 weeks old) were purchased from Envigo (Gannat, France) and housed in an SPF facility. A total of 3×10^5^ B16-OVA cells were implanted s.c into the right thigh of mice that were sedated with 2.5% isoflurane and 1 L.min^-1^ oxygen flow rate (Abbott). Monitoring of tumor growth was performed daily. A caliper was used to measure the dimensions (length and width) of palpable tumors. Mice received a dose of 10 µg fLuc mRNA DiR-labeled Galsomes (high dose) in a total volume of 200 μl via i.v. injection in the tail vein 11 days after inoculation. 24 h after the injection, mice were anesthetized in a ventilated anesthesia chamber with 3% isoflurane in oxygen, and the chest, abdomen and right hind leg were depilated with hair removal cream. Subsequently, VivoGlo Luciferin (Promega) was administered intraperitoneally (i.p.). After 10 min, bioluminescence and fluorescence images were acquired by the IVIS lumina II system (PerkinElmer, Waltham, MA, USA). Images were quantitatively analysed using LivingImage software (PerkinElmer).

### Labeling of nanobodies with ^99m^Tc-pinhole SPECT/microCT imaging and analysis

Repeated non-invasive imaging of PD-L1 was performed on days 1, 4, 7, 10 and 14 after treatment of B16-OVA-bearing mice with PBS or Galsomes. Nanobody C3 that specifically binds mouse PD-L1 was coupled via its carboxyl-terminal histidine (HIS_6_)-tag with a ^99m^Tc-tricarbonyl intermediate [^99m^Tc][Tc(H_2_O)_3_(CO)_3_]^+^ that was synthesized using the Isolink® labeling kit (Mallinckrodt Medical BV) 20. The solution of radiolabeled nanobodies was purified on a NAP5 column (GE Healthcare) pre-equilibrated with PBS to remove unbound [^99m^Tc][Tc(H_2_O)_3_(CO)_3_]^+^. Filtration over a 0.22 μm filter (Millipore) was performed to remove aggregates. The labeling efficiency was determined directly after labeling and after purification by radioactive instant thin layer chromatography with 100% acetone as the mobile phase. Mice were i.v. injected with radiolabeled nanobodies with a radiochemical purity of >95% and a specific activity of 12.5±2.2 MBq/µg and on average 62.4±11.0 MBq of injected activity. Pinhole SPECT/microCT imaging was performed on anesthetized mice (Ketamidor, Richter Pharma AG) using a Vector+/CT scanner (MILabs). Image reconstruction was performed after which 15×18×17 mm³, 3.5×2.5×3.5 mm³ or 7×4×6 mm³ ellipsoid regions of interests (ROIs) were positioned on tumor tissue, lungs and liver, respectively to quantify uptake of the radiolabeled nanobodies. For lung and liver uptake concentration, a smaller ROI was positioned within the organ, and the relative uptake concentration was obtained directly from the imposed ROI in the AMIDE imaging software (Medical Image Data Examiner software, version 1.0.4) using the injected activity and the camera calibration factor as input parameters. Tumor uptake was calculated by the use of a large ROI positioned over the total tumor and surrounding non-tissue containing areas, and subsequently dividing total activity by the callipered tumor volume. The results obtained in AMIDE were corrected for decay, calculated and expressed as percentage of injected activity per cubic centimeter of callipered tumor volume (%IA/cc). Representative SPECT/CT images are overlays of SPECT and CT signals and were generated using three-dimensional maximum intensity projection (3D MIP) SPECT.

### Preparation of single cell suspensions from organs

Single cell suspensions were prepared from lungs, liver, and tumor. Lungs, liver, and tumor were digested using enzymatic digestion kits and an OctoMACS tissue dissociator (Miltenyi Biotec) according to the manufacturer's specifications. Red blood cells were removed by addition of a red blood cell lysis buffer (Biolegend).

### Flow cytometry

To study expression of MHC-I (H2-K^b^) and MHC-I/OVA (H2-K^b^/SIINFEKL) complexes on B16 and B16-OVA cells, cultured for 24 h in the absence or presence of 50 ng recombinant murine IFN-γ (Immunotools), cells were stained with a FITC-conjugated anti-H2-K^b^ antibody (clone AF6-88.5.5.3, eBioscience) or an APC-conjugated anti-H2-K^b^/SIINFEKL antibody (clone 25-D1.16, RRID: AB_11219595). Cells were acquired on the LSR Fortessa and analyzed using FACS Diva software (Becton Dickinson [BD]). Gating of viable cells was performed on FSC/SSC characteristics, unstained cells were used to gate for MHC-I positivity, while B16 cells were used to gate for H2-K^b^/SIINFEKL positivity.

Analysis of CD8^+^ T cells (OT-I) that produce IFN-γ upon recognition of H2-K^b^/SIINFEKL by their T cell receptor (TCR) was performed by staining the T cells with a PerCP-Cy5.5-conjugated anti-CD8α antibody (clone 53-6.7, RRID: AB_394081) after which the cells were fixed and permeabilized using the Cytofix/Cytoperm kit (BD) for intracellular staining of IFN-γ by the APC-conjugated anti-IFN-γ antibody (clone 554413, RRID: AB_398551). Cells were acquired on the LSR Fortessa and analyzed with FlowJo™ software (BD, RRID:SCR_008520). Gating of viable cells was performed on FSC/SSC characteristics, unstained cells were used to gate for CD8 positivity, while unstimulated CD8^+^ T cells were used to gate for IFN-γ positivity.

Single cell suspensions were used to analyse the immune cell composition and expression of PD-1 and PD-L1 in selected organs. Cells were incubated with anti-CD16/32 antibodies (Biolegend, RRID: AB_312801) and stained with Live/Dead marker (LIVE/DEAD™ Fixable Green Dead Cell Stain Kit, Thermo Fisher Scientific). The following antibodies were used to stain (*i*) conventional T cells: anti-CD45.2-APC-eF780 (clone 104, RRID:AB_1727492), anti-CD3ε-BV605 (clone 145-2C11, RRID: AB_2737945), anti-CD4-AF700 (clone RM4-5, RRID: AB_396956), anti-CD8α-PerCP-Cy5.5 (clone, 53-6.7, RRID: AB_394081), and anti-PD-L1-BV421 (clone MIH5, RRID:AB_2738911); (*ii*) myeloid cells: anti-CD45.2-APC-eF780 (clone 104, RRID: AB_1727492), anti-CD11b-AF700 (clone M1/70, RRID: AB_396960), anti-Ly6G-AF647 (clone 1A8, RRID: AB_1727560), anti-MHC-II-PE/Dazzle594 (clone M5/114.15.2, RRID: AB_2565979), anti-CD11c-BV650 (clone HL3, RRID: AB_2725779), anti-F4/80-BB700 (clone T45-2342, RRID:AB_2743450), and anti-PD-L1-BV421; (*iii*) iNKT cells: anti-CD45.2-APC-eF780, anti-TCRβ-APC (clone H57-597). All antibodies were obtained from BD except for anti-MHC-II antibodies that were obtained from Biolegend. To study iNKT cells and OVA-specific CD8^+^ T cells, samples were additionally stained with PE-labeled mCD1d-PBS57 tetramer (kindly provided by the NIH tetramer core facility) and APC-conjugated OVA dextramer (Immudex), respectively. Cells were acquired on a FACS Celesta and analyzed using FlowJo™ software (BD, RRID: SCR_008520). Gating strategy is shown in Figure S1.

### Statistical analysis

Kaplan-Meier curves that show the time-to-reach humane endpoints were statistically analysed using Log-Rank (Mantel-Cox) tests. All other statistical tests are described in figure legends. All statistical analyses, except for statistical prediction models, were conducted in Graphpad™ Prism 9.3.1. The identification and figures for the regression models were developed using the Linear and Nonlinear Mixed effects Models 27 and Graphics for Data Analysis 28 in software R (R Core Team 2021). Given the no linear behavior of PD-L1 along time for the different conditions (PBS, low and high doses) the regression models were done over the transformed data 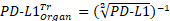. Respectively, the obtained regression models for the PD-L1 signal in lung, liver and tumor are:


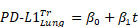
+ 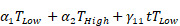
+ 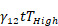



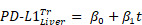
+ 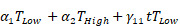
+ 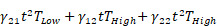



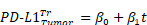
+ 
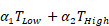


With t the days after vaccination, 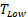 the low αGC-dose and 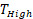 the high αGC-dose.

The number of times experiments were repeated (n) as well as the number of mice per condition (mpc) are indicated in the figure legends. The asterisks in the figures indicate the level of statistical significance: *, p<0.05; **, p<0.01, ***, p<0.001, ****, p<0.0001.

## Results

### Galsome vaccination induces an αGC-dose-dependent immune response

**Figure 1:**
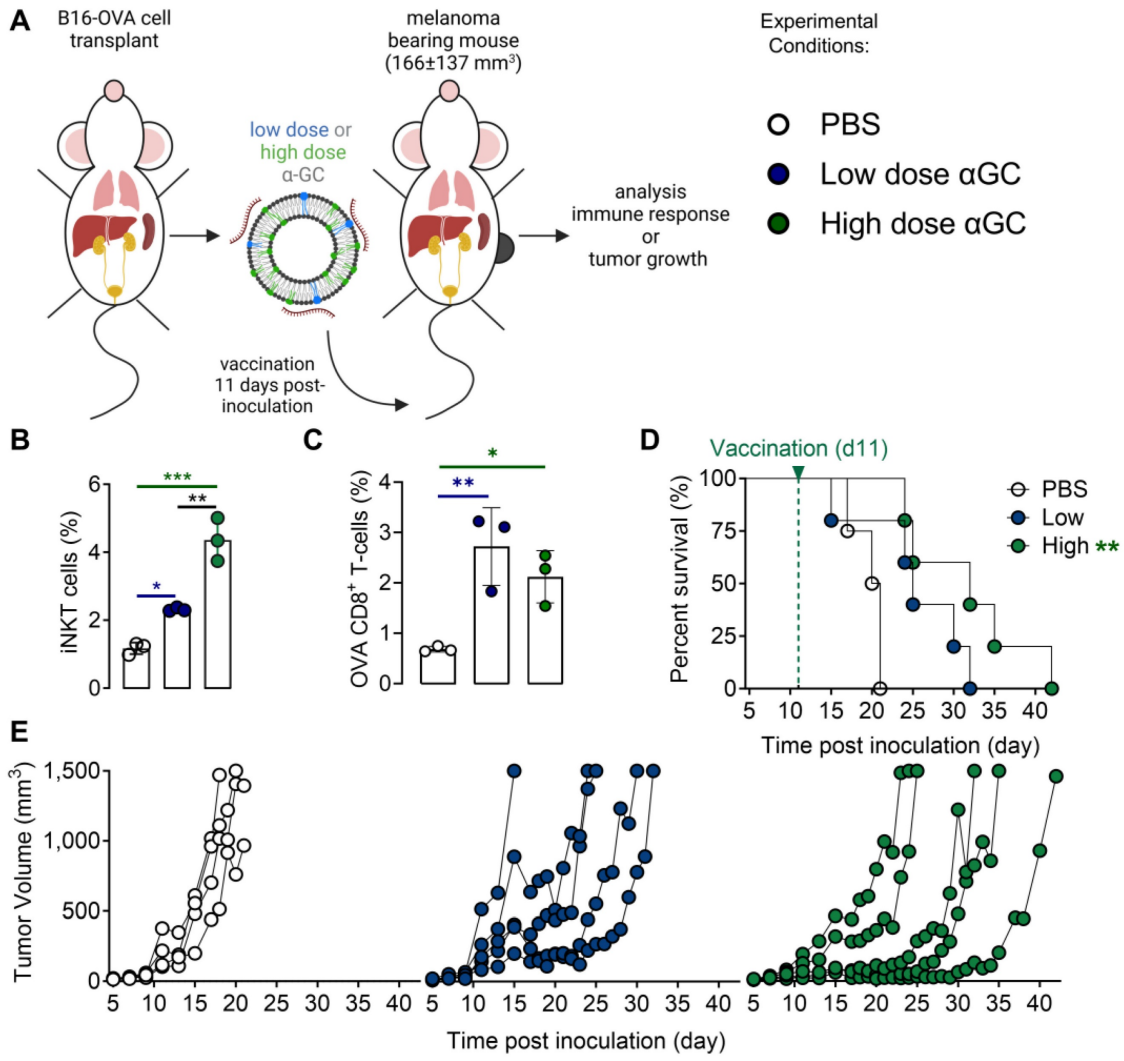
** Galsome vaccination induces αGC-dose-dependent therapeutic anti-melanoma immune responses. (A)** Schematic overview of the Galsome vaccination regimen. **(B-C)** Mean percentage ± SD of **(B)** iNKT cells and **(C)** OVA-specific CD8^+^ T cells detected in the spleen 4 days after vaccination within the CD45^+^ and CD45^+^CD3^+^ populations respectively. Symbols represent individual data points. Statistical analysis was performed by one-way ANOVA with Holm-Sidak's multiple comparison test (n=1, mpc=3).** (D)** Kaplan-Meier plot showing the time to reach humane endpoints and statistically analysed using Log-Rank (Mantel-Cox) tests. **(E)** Tumor growth kinetics showing the progression of tumors per mouse until day 42. The data are representative of one out of 2 independent experiments (n=2, mpc=4-6).

A therapeutic vaccination study with OVA-encoding mRNA Galsomes in an OVA-expressing melanoma tumor model was performed (Figure 1A). To this end, B16-OVA cells were generated through the transduction of melanoma B16 cells with lentiviral vectors encoding OVA (B16-OVA), which allowed to evaluate therapy response to Galsome-induced OVA-specific T cell responses as shown in Figure S2. To study the impact of the αGC-dose on the induction of therapeutic immune responses, we prepared two Galsome-formulations containing a fixed dose of 10 µg OVA mRNA and either a low (2 ng) or high (20 ng) dose of αGC. Low and high doses were selected based on an earlier study performed by Verbeke et al. making use of mRNA-Galsomes with the high αGC-dose (20ng) 11 Modified mRNA (1-mѰU) was used to achieve maximal mRNA expression and to assure a high antigen presentation to CD8^+^ T cells. We showed that the αGC-dose did not impact the size, polydispersity, and charge of mRNA Galsomes (Figure S3). Subsequently, C57BL/6 mice were subcutaneously (s.c.) implanted with a B16-OVA tumor and vaccinated with two different Galsome formulations (low and high αGC-dose) or PBS when tumors reached a size of 166±137 mm^3^ (11 days post inoculation), as shown in Figure 1A. The immune response was evaluated by measuring the number of anti-tumor immune effector cells (iNKT cells and OVA-specific CD8^+^ T cells) in the spleen 4 days after vaccination, showing a significant increase in iNKT cells and OVA-specific CD8^+^ T cells upon vaccination of both αGC-doses (Figure 1B-C). We also studied the therapeutic outcome by monitoring tumor growth and registering the time-to-reach humane endpoints, *i.e.,* a tumor volume of 1500 mm^3^ or weight loss exceeding 20% of initial weight. Mice vaccinated with Galsomes containing a low or high dose of αGC showed a delay in tumor growth resulting in an increased time to reach humane endpoints (Figure 1D-E). Compared to mice treated with PBS, only mice vaccinated with the high aGC-Galsomes showed a significant therapeutic effect (*p= 0.005062*), although a dose-dependent delay in tumor growth was observed.

### PD-L1 is rapidly and systemically upregulated after Galsome vaccination

**Figure 2:**
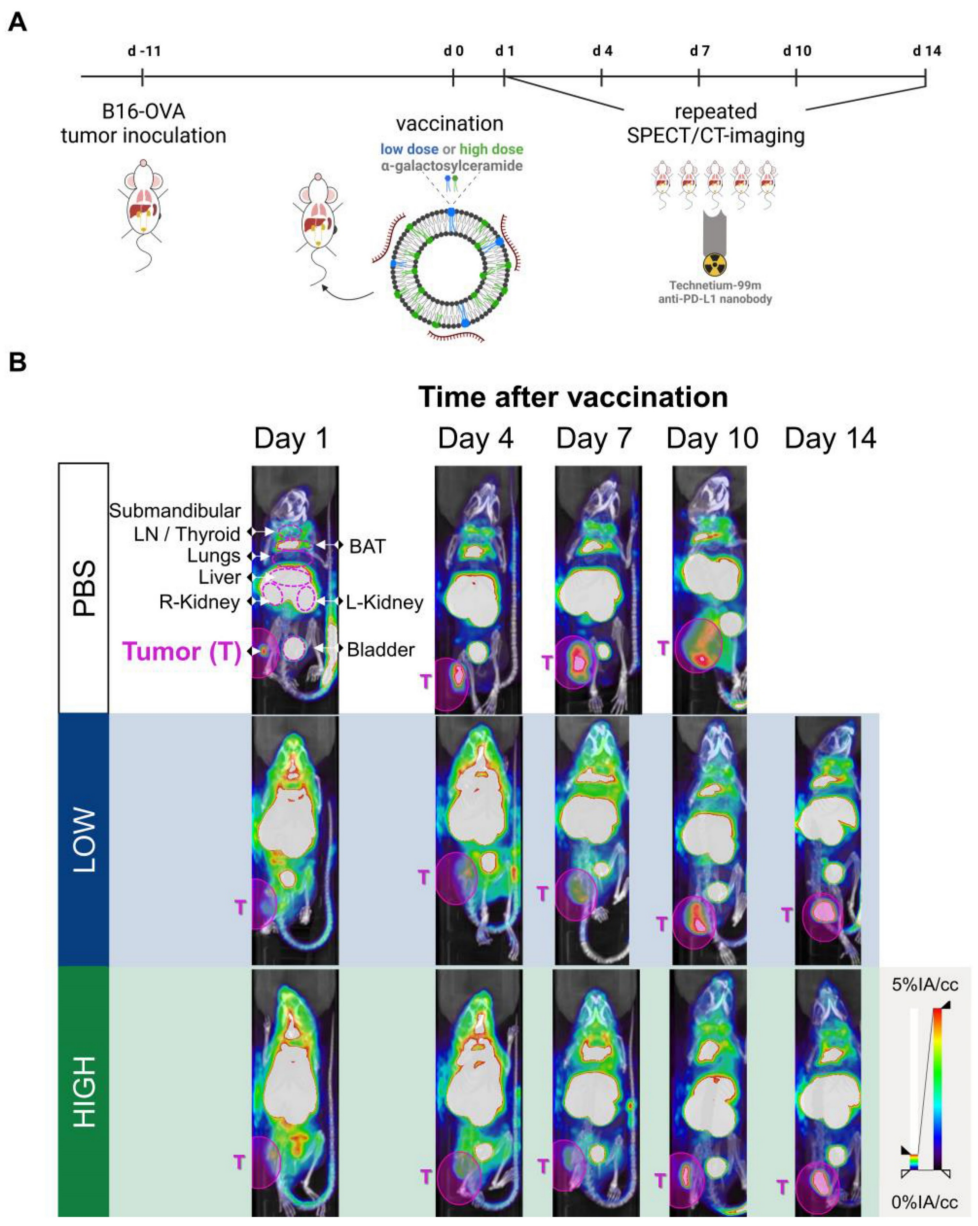
** Galsome vaccination alters PD-L1 expression. (A)** Schematic overview of the in vivo imaging experiment.** (B)** Representative coronal view images of 3D MIP SPECT/microCT scans showing the accumulation of anti-PD-L1 ^99m^Tc-nanobodies in B16-OVA-bearing mice that were treated 1, 4, 7, 10 or 14 days prior with PBS or Galsomes containing a low or a high αGC-dose (n=2, mpc=4-5). BAT = Brown adipose tissue, LN = Lymph node.

Both iNKT cells and CD8^+^ T cells that are activated upon Galsome vaccination produce IFN-γ, a driving force for PD-L1 expression 29,30. Therefore, PD-L1 expression is expected to be upregulated upon vaccination. We studied the spatiotemporal expression of PD-L1 by serial SPECT/microCT imaging on days 1, 4, 7, 10 and 14 after injection of mice with Galsomes or PBS as schematically represented in Figure 2A. Since previous results showed a dose-dependent proliferation of iNKT cells (Figure 1B), we studied the effect of two αGC-doses on PD-L1 upregulation. As a tracer for PD-L1 detection we used a nanobody that we previously developed and specifically binds mouse PD-L1 23.

We showed that Galsome vaccination, irrespective of the dose of αGC, induced a strong systemic upregulation of PD-L1 over a period of several days compared to untreated mice (Figure 2B). A high accumulation of the ^99m^Tc-labeled PD-L1-specific nanobody was observed in kidneys and bladder in all mice (Figure 2B), which was expected as nanobodies are filtered from the system via the kidneys due to their small size (13kDa). Furthermore, a high accumulation of the anti-PD-L1 nanobody was observed in brown adipose tissue (BAT), lymph nodes and liver of both untreated and treated mice (Figure 2B), which is in line with the anti-PD-L1 nanobody distribution described in earlier experiments 23. BAT and lymph nodes were shown to have a high basal PD-L1 expression which corresponds with the high accumulation of ^99m^Tc-nanobodies. The large activity deposit in liver has been partially ascribed due to non-specific uptake of this nanobody 23 Noteworthy, the bladder of vaccinated mice contained less activity compared to untreated mice (PBS) 1 day upon vaccination, highlighting the high systemic uptake of the radioactive tracer in treated mice.

**Figure 3:**
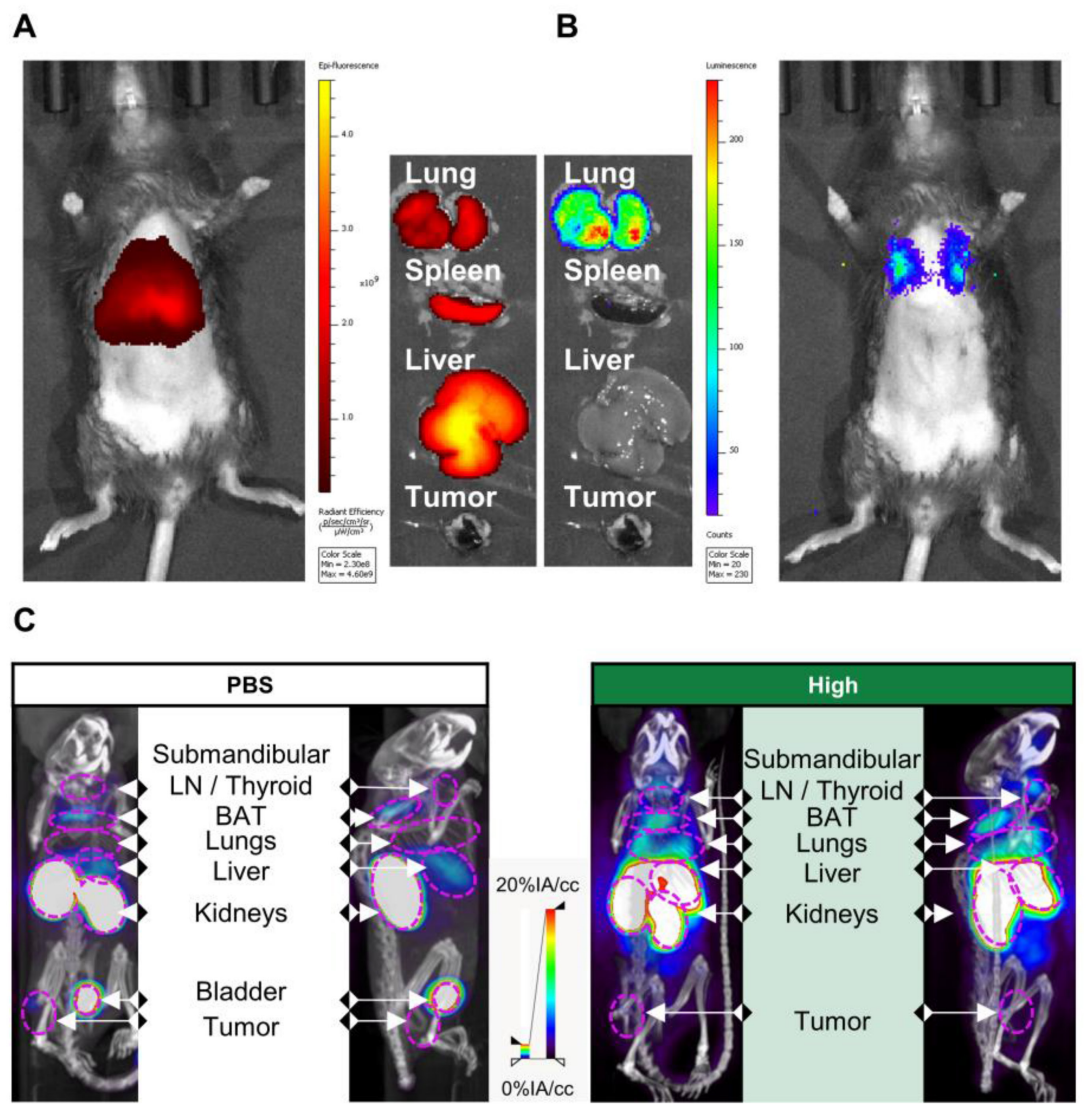
** PD-L1 expression 24 h after vaccination in part overlaps with the biodistribution of Galsomes. (A, B)** Representative image of** (A)** Galsome distribution using near-infrared fluorescence and **(B)** bioluminescence in B16-OVA-tumor bearing mice 24 h after administration of DiR-loaded Galsome particles complexed with fLuc mRNA.** (C)** Representative image of systemic anti-PD-L1 ^99m^Tc-nanobody distribution 1 hour post injection via 3D MIP SPECT/CT overlay (coronal [left] and sagittal [right] view) 24 h after administration of PBS or Galsomes containing a high αGC-dose (High) in B16-OVA bearing mice. BAT = Brown adipose tissue, LN = Lymph node.

To link these PD-L1 expression maps to mRNA Galsomes biodistribution, we labeled Galsomes, containing mRNA encoding fLuc, with the lipophilic dye DiR. This allowed us to visualize both the biodistribution of the mRNA Galsomes and fLuc expression with whole animal imaging. Figure 3A-B shows that DiR-loaded Galsomes strongly accumulated in lung, spleen and liver while mRNA expression was limited to lungs 24 h post vaccination 11,31. This discrepancy in mRNA lipid nanoparticle biodistribution and expression of mRNA has been observed by other authors and is possibly a consequence of the uptake of mRNA lipid by different cell types 32. Interestingly, analogous to the biodistribution of Galsomes, lungs and liver displayed an increased radiotracer uptake one day after administration of Galsomes containing a high αGC-dose compared to untreated mice (PBS), indicating increased PD-L1 expression in these organs where the vaccine accumulated (Figure 3C). Due to the small anatomical distance between spleen and kidney, it was impossible to quantify the radiotracer uptake in the spleen.

### PD-L1 radiotracer uptake kinetics in lungs and liver differ but drop to baseline levels a few days after vaccination

**Figure 4:**
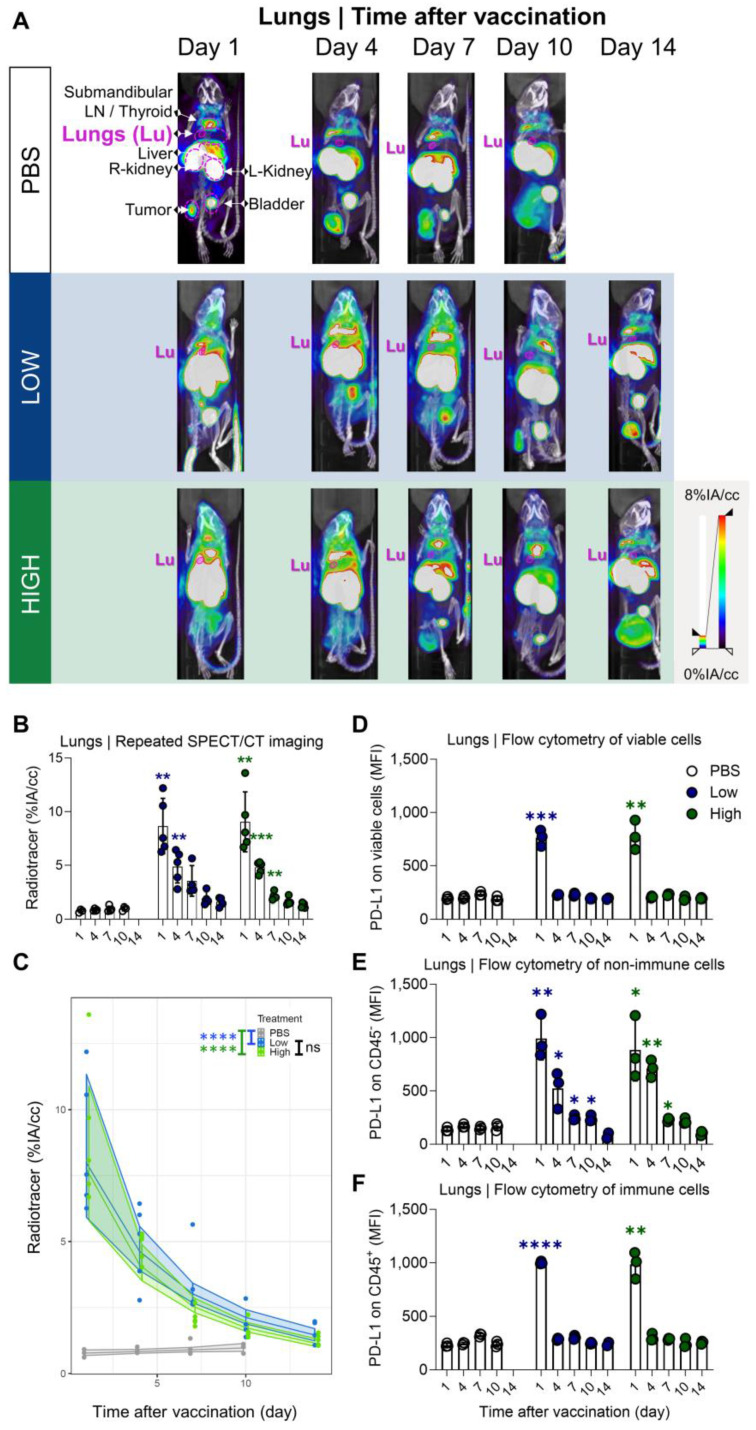
** Quantification of radiotracer uptake in lungs shows a gradual decrease after vaccination and corresponds with PD-L1 expression on non-immune cells in flow cytometry. (A)** Representative 3D MIP SPECT/microCT images (coronal view) showing the systemic accumulation of anti-PD-L1 ^99m^Tc-nanobodies. B16-OVA-bearing mice were imaged 1, 4, 7, 10 or 14 days after treatment with PBS or Galsomes containing a low or a high dose αGC. **(B)** Radiotracer accumulation in lung tissue, as quantified by imaging. Data are shown as mean ± SD of one out of two independent experiments. Statistical analysis was performed by unpaired multiple t-test with Welch correction (n=2, mpc=4-5). **(C)** Regression model with confidence intervals and raw data points. The curve and the intervals are obtained by inverse transformation of the proposed model (see statistical analysis and Tables S1 and S2). Flow cytometry results represented as mean fluorescence intensity (MFI) of the viable **(D)**, non-immune (CD45^-^) **(E)** and immune (CD45^+^) **(F)** cell population in lung tissue (n=1, mpc=3). Data are shown as mean ± SD. Statistical analysis was performed by unpaired multiple t-tests. Lu = Lungs, LN = Lymph node.

**Figure 5:**
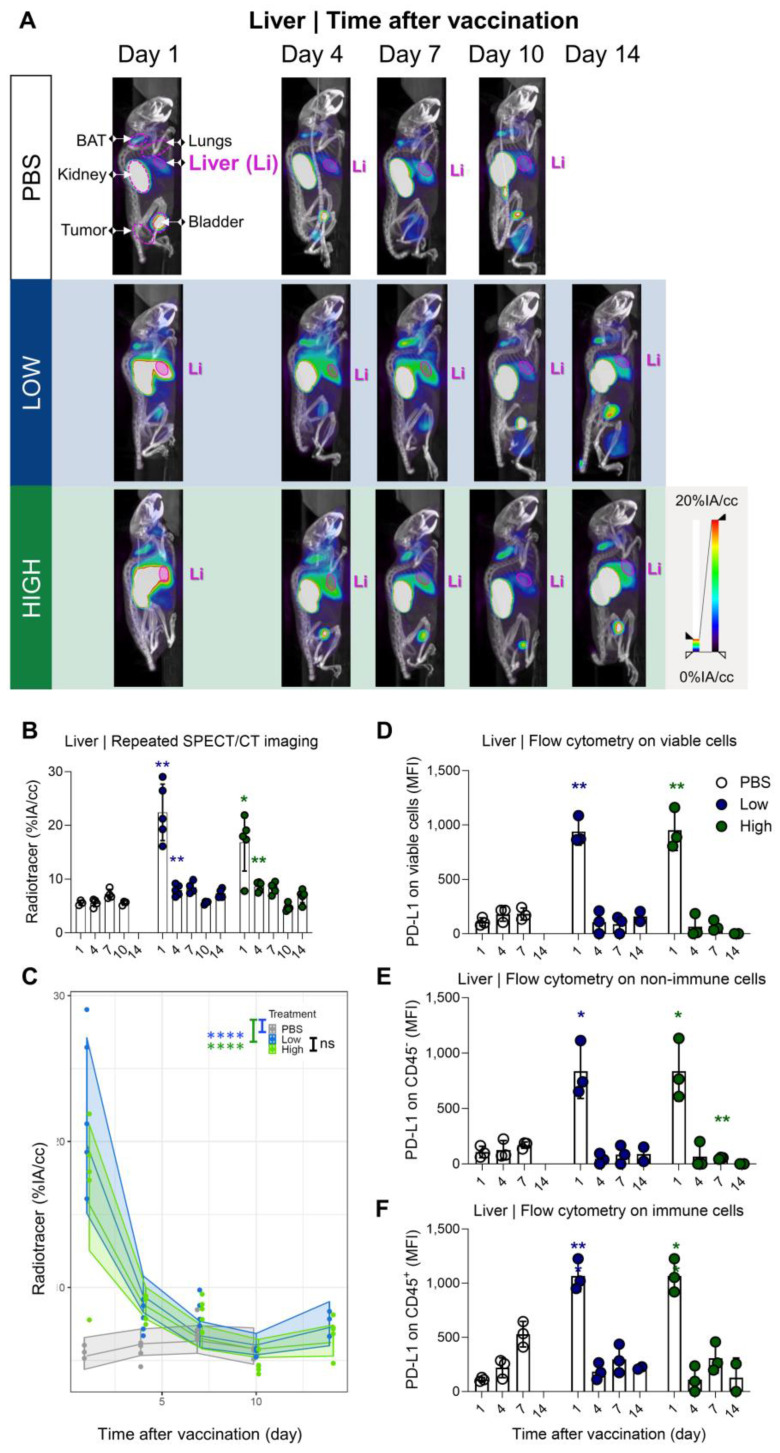
** Galsome vaccination induces rapid, short-lasting expression of PD-L1 in liver. (A)** Representative 3D MIP SPECT/microCT images (sagittal view) showing the whole-body accumulation of anti-PD-L1 ^99m^Tc-nanobodies in chronological order after vaccination. B16-OVA-bearing mice were treated 1, 4, 7, 10 or 14 days prior with PBS or Galsomes containing a low or a high αGC-dose (mpc=4-5). (B) Radiotracer accumulation in liver tissue, as quantified by imaging. Data are shown as mean ± SD of one out of two independent experiments. Statistical analysis was performed by unpaired multiple t-test with Welch correction (n=2, mpc=4-5). (C) Regression model with confidence intervals and raw data points. The curve and the intervals are obtained by inverse transformation of the proposed model (see statistical analysis and Tables S3 and S4). Flow cytometry results represented as MFI of the viable (D), non-immune (CD45-) (E) and immune (CD45+) (F) cell population in liver tissue. (n=1, mpc=3) Data are shown as mean ± SD. Statistical analysis was performed by unpaired multiple t-test. Li = Liver, LN = Lymph node.)

Our results showed a strong and rapid upregulation of PD-L1 after vaccination, which was most pronounced in liver and lung and corresponded with Galsome biodistribution. Therefore, we decided to further analyse the upregulation of PD-L1 by positioning ROIs over these organs and quantifying the accumulation of the ^99m^Tc-labeled PD-L1-specific nanobody as a percentage of injected activity over tissue volume. We showed that PD-L1 expression was strongly upregulated in both organs (liver and lung) as early as one day after vaccination (Figure 4A-B and 5A-B). PD-L1 expression levels gradually decreased to baseline over a period of 7 days in the lung (Figure 4A-B), whereas PD-L1 expression in the liver already receded to baseline levels by the next time point of the analysis (4 days after vaccination) (Figure 5A-B). Subsequently, a statistical regression model was applied to evaluate if PD-L1 is dose-dependently upregulated in vaccinated mice and if there is a time-dependent effect on the expression levels (Figure 4C and 5C and Tables S1-4). The regression model indicated that PD-L1 expression in liver and lung of control mice did not significantly change over time, while it was significantly upregulated in liver and lungs of vaccinated mice. However, no significant differences in PD-L1 were found between the two αGC-doses in Galsomes (Tables S2 and S4).

To corroborate the observations obtained with SPECT/microCT imaging at the single cell level, additional mice were included in the study and sacrificed on days 1, 4, 7, 10 and 14. Lung and liver tissue were processed to single cell suspensions and analysed with flow cytometry. Strikingly, we observed clear differences between the data obtained with SPECT/CT imaging and flow cytometry (Figure 4D). This could possibly be attributed to the organ digestion protocol, which focused on the conservation of immune cells and consequently led to selective enrichment of these cells, while partly losing non-immune cells which make up the majority of the total lung (*e.g.*, 89% epithelial cells) 33. Therefore, we analysed the PD-L1 expression within the immune (CD45^+^) subpopulation and non-immune (CD45^-^) subpopulation, which represented 90% and 10% of the viable population respectively. Indeed, while PD-L1 expression in immune cells (Figure 4F) showed a rapid PD-L1 upregulation and subsequent downregulation, a different PD-L1 expression pattern was observed for non-immune cells (Figure 4E). Here, a rapid PD-L1 upregulation was followed by a gradual decrease in PD-L1 expression as a function of time consistent with the whole-body imaging data. These findings suggest that in the lungs, non-immune cells contribute to PD-L1 expression after vaccination to a larger extent which dominates the whole body images. In the liver, both, non-immune (Figure 5E) and immune cells (Figure 5F) showed the same PD-L1 expression pattern as the whole viable cell population (Figure 5D), which corresponded to the whole-body imaging results. For both organs, we also evaluated the number of PD-L1^+^ cells in these cell populations as this obviously also contributes to the overall PD-L1 expression. (Figure S4A-F) shows that both variables (MFI of PD-L1^+^ cells and amount of PD-L1^+^ cells) showed the same trends strengthening our conclusions.

### Myeloid cells as well as non-immune cells contribute to vaccination-induced upregulation of PD-L1 in liver and lungs

**Figure 6:**
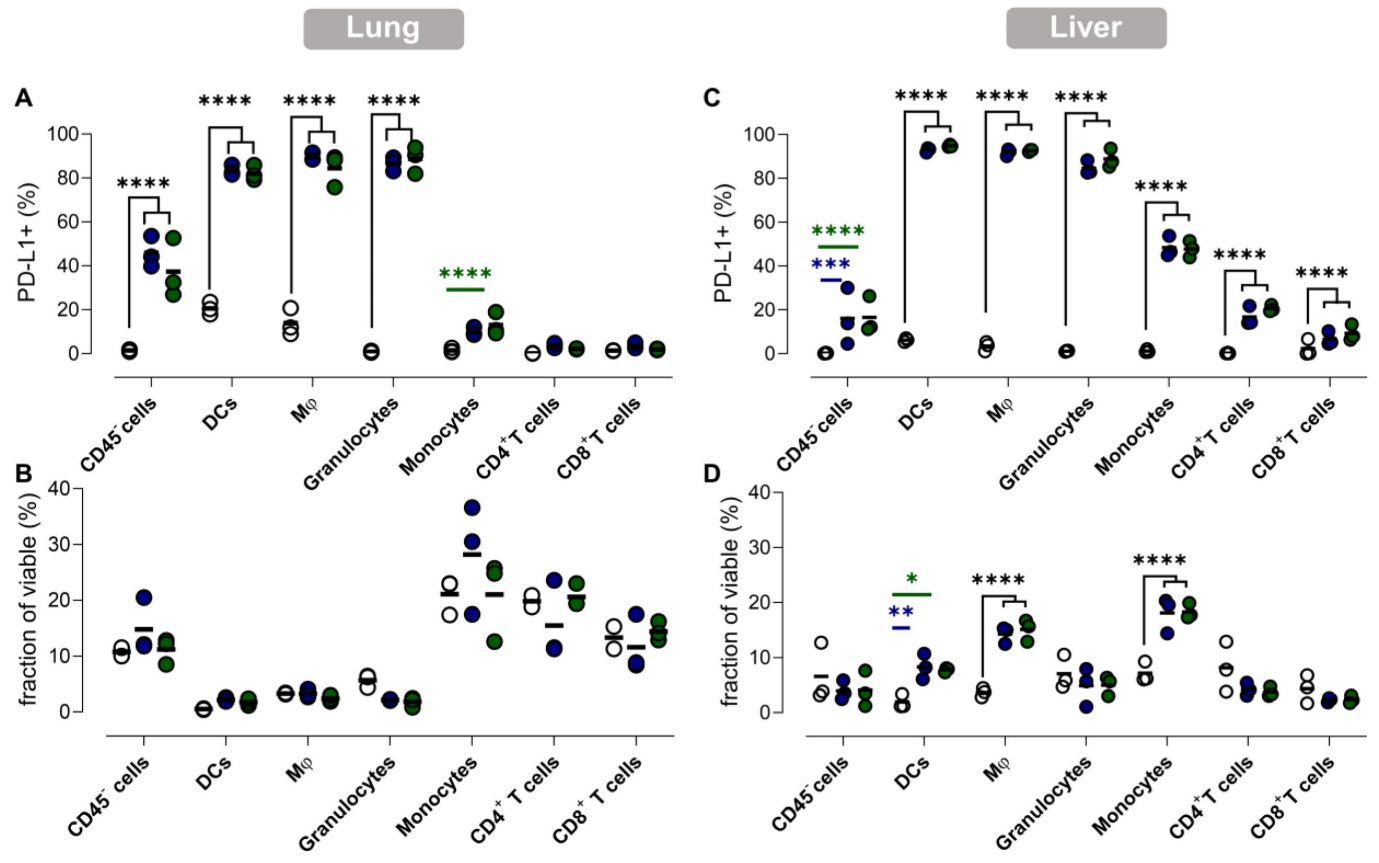
** PD-L1 expression in lung and liver is elevated in myeloid cells and non-immune cells but also in T cells in liver. (A,C)** The fraction of PD-L1^+^ non-immune cells (CD45^-^), DCs, macrophages (Mφ), granulocytes, monocytes, CD4^+^ and CD8^+^ T cells 1 day after vaccination with Galsomes containing a low (blue) or high (green) αGC-dose or PBS (grey) in (A) lung and (C) liver. (B,D) The fraction of non-immune cells and different immune cell types within viable cells 1 day after vaccination in (B) lung and (D) liver. Symbols represent individual data points and mean is indicated by a line. Statistical analysis was performed by two-way ANOVA followed by Tukey's post hoc test. (n=1, mpc=3).

To gain more insight into the effect of vaccination on PD-L1 expression at the cellular level, we performed flow cytometry analysis of different immune cell types in lung and liver tissue. This analysis was performed 1 day after vaccination, as for both organs the highest PD-L1 expression levels were measured at this timepoint. We studied the prevalence and the number of PD-L1 expressing cells of several immune cell types and non-immune (CD45^-^) cells in the lung and liver (Figure 6).

In accordance with the previous observations, dynamics of PD-L1 expression in lungs and liver were dissimilar. We observed a drastic upregulation of PD-L1 on non-immune and myeloid cells in lung tissue after vaccination (Figure 6A), while no significant influx of immune cells or other cell types in lungs was observed (Figure 6B). In contrast, we observed a significant increase of myeloid cells (except granulocytes) in the liver of vaccinated mice, which coincided with a rising fraction of PD-L1^+^ myeloid cells (Figure 6D). Remarkably, PD-L1 was also upregulated on T cells in the liver (Figure 6C). Note that increasing the αGC-dose from low to high did not result in a significant increase of PD-L1^+^ cells or in a higher influx of immune cells one day after vaccination.

The high radiotracer uptake observed in liver and lung by *in vivo* imaging combined with the uptake of Galsomes in the liver and lung, made us question whether this PD-L1 expression could be the result of activation and thus presence of iNKT cells and OVA-specific CD8^+^ T cells 31. Lung tissue was of special interest since we also observed transfection in this organ, which is crucial for antigen presentation. We showed a significantly higher number of OVA-specific CD8^+^ T cells in the lung, which peaked 4 to 10 days after Galsome treatment (Figure. S5A). iNKT cells were peaking at an earlier timepoint, more specifically 4 days after vaccination with a high αGC-dose Galsomes (Figure S5B). In liver, similar kinetics of OVA-specific CD8^+^ T cells and iNKT cells were observed upon vaccination, albeit less pronounced (Figure S5C and D). As we observed a discrepancy between cellular responses and PD-L1 expression, these data suggest that PD-L1 expression and effector cell proliferation were not correlated.

### Administration of Galsomes with a high dose of αGC coincides with a modest and transiently elevated expression of PD-L1 in tumors

**Figure 7:**
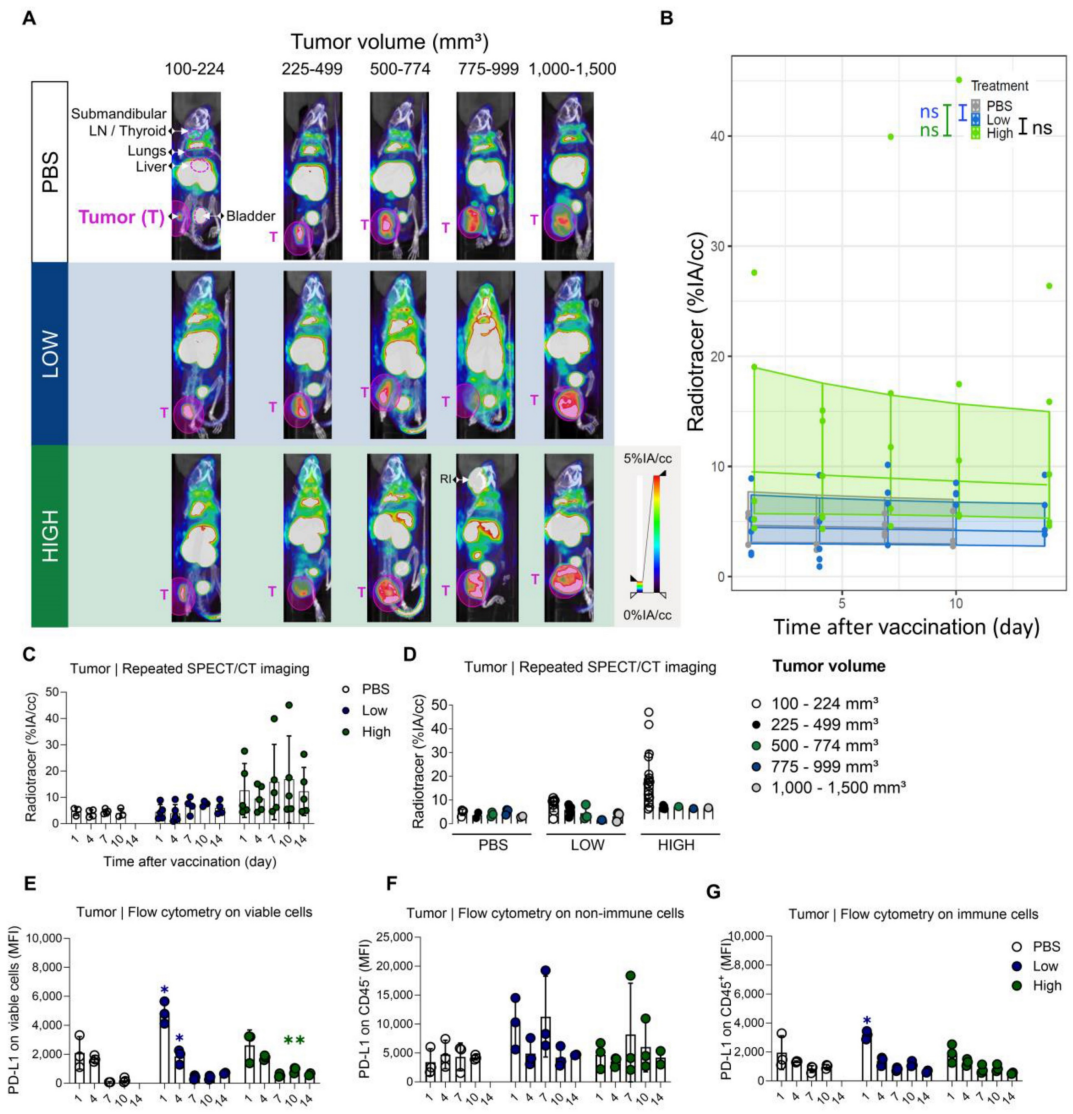
** An elevated PD-L1 expression in tumors of mice upon vaccination with Galsomes. (A)** Representative 3D MIP SPECT/microCT images (coronal view) showing the whole-body accumulation of anti-PD-L1 ^99m^Tc-nanobodies. Images were stratified in function of tumor volume. B16-OVA bearing mice were treated 1, 4, 7, 10 or 14 days prior with PBS or Galsomes containing a low or a high αGC-dose (n=1, mpc=4-5). **(B)** Regression model with confidence intervals and raw data points. The curve and the intervals are obtained by inverse transformation of the proposed model (see statistical analysis and Tables S5 and S6)***.* (C,D)** Radiotracer accumulation in tumor tissue, as quantified by imaging. Data are shown as mean ± SD and displayed in function of (C) time and (D) tumor size. Symbols represent individual data points. This confidence intervals are indicative. Flow cytometry results represented as MFI of the viable **(E)**, non-immune (CD45^-^) **(F)** and immune (CD45^+^) **(G)** cell population in tumor tissue (n=1, mpc=3). Data are shown as mean ± SD. Statistical analysis was performed by unpaired multiple t-test. T = Tumor, LN = Lymph node, RI = Retro-orbital injection of ^99m^Tc-anti-PD-L1 nanobodies (upon incomplete tail vein injection).

Evidently, we were also interested in the time-dependent PD-L1 expression in the tumor after vaccination. Therefore, SPECT/microCT images obtained on days 1, 4, 7, 10, and 14 after Galsomes vaccination were used to quantify the upregulation of PD-L1 in tumors using ROIs positioned over the entire tumor and compared to control mice. Tumor uptake was analysed over time and in accordance with tumor size as this can also influence PD-L1 expression and as such represent a confounding factor. We observed that as tumors grew, the accumulation of the PD-L1 radiotracer was visibly increased upon vaccination yet displayed a heterogenous distribution in tumor tissue on the SPECT/microCT images (Figure 7A-C). However, when we corrected for tumor volume by quantifying the accumulation of the PD-L1 radiotracer per calipered tumor volume (cc), we observed that small tumors in mice treated with Galsomes containing a high αGC-dose showed the highest levels of PD-L1 tracer uptake (Figure 7D). The regression model did not detect any change in PD-L1 radiotracer uptake upon vaccination, despite the p-value approximating 0.05 and curves showing an elevated PD-L1 radiotracer uptake for mice treated with Galsomes containing a high αGC-dose (Figure 7B, Tables S5 and S6). In addition, a moderate time-dependency of PD-L1 expression was observed for mice treated with Galsomes containing a high dose αGC, but not for mice treated with a low dose and PBS (Table S6). This is in stark contrast with flow cytometry data, displaying a significantly increased expression of PD-L1 in the tumor of mice injected with Galsomes with a low αGC-dose on day 1 after vaccination Figure 7E-G).

### The tumor microenvironment shows high baseline PD-L1 expression which is shifted in various immune cell populations in response to vaccination

Analogous to lung and liver, we analysed the cellular composition of the tumor and evaluated PD-L1 expression of the individual cell types with flow cytometry 1 and 7 days after vaccination with PBS or Galsomes.

**Figure 8:**
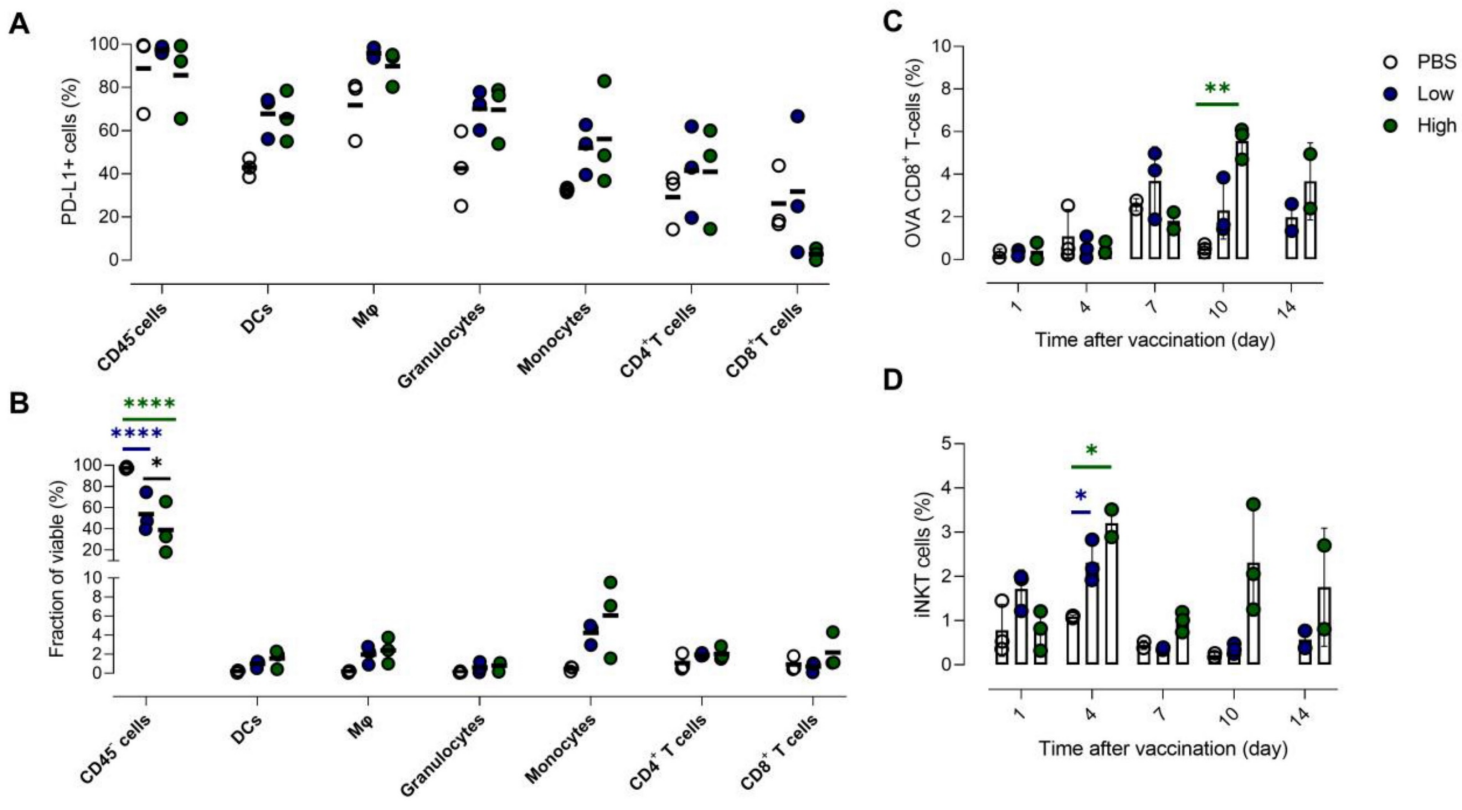
** High baseline expression of PD-L1 in tumor is slightly increased in various immune cell populations upon Galsome vaccination. (A)** Fraction of PD-L1^+^ non-immune (CD45^-^) cells, DCs, macrophages (Mφ), granulocytes, monocytes, CD4^+^ and CD8^+^ T cells 7 days after vaccination with Galsomes containing a low (blue) or high (green) αGC-dose or PBS (white) in tumor. **(B)** Fraction of non-immune cells and different immune cell types within viable cells 7 days after vaccination. **(C, D)** Percentage of OVA-specific CD8^+^ T cells (C) and iNKT cells (D) within the CD45^+^ population in the tumor detected on days 1, 4, 7, 10 and 14 after vaccination. Symbols represent individual data points and mean is indicated by a line. Statistical analysis was performed by two-way ANOVA followed by Tukey's post hoc test. (n=1, mpc=3).

One day after vaccination, we could only detect a significantly higher PD-L1 expression in granulocytes (Figure S6A). However, in the remaining immune cell types (myeloid cells and CD4^+^ T cells) we also noticed a trend of elevated PD-L1 expression. In addition, we observed a slight reduction of the myeloid cell fraction in the TME 1 day after vaccination (Figure S6B) and a seemingly increased number of non-immune cells, which is a consequence of a shift in the balance of both cell types. Interestingly, we observed a dose-dependent influx of monocytes 7 days after vaccination (Figure 8B). All myeloid cell types that were analysed showed an increasing trend of PD-L1 expression (Figure 8A). We also addressed whether PD-L1 expression in tumors coincided with infiltration of iNKT cells and OVA-specific CD8^+^ T cells. We showed that mice treated with Galsomes had a higher percentage of OVA-specific CD8^+^ T cells 10 days after vaccination (Figure 8C). In contrast to peripheral organs, only a high αGC-dose was sufficient to recruit a significantly increased amount of OVA-specific CD8^+^ T cells to the tumor. We also showed that the percentage of iNKT cells in mice treated with Galsomes containing a high αGC-dose increased by day 4 yet failed to further increase over time (Figure 8D). As also seen in liver and lung, we did not observe a correlation between the number of OVA-specific CD8^+^ T cells or iNKT cells in the tumor and the image-based PD-L1 expression in tumors.

## Discussion

Designing vaccines that allow *in situ* activation of the immune system has been a major focus in cancer immunotherapy. We previously developed mRNA Galsomes, an mRNA cancer vaccine consisting of a lipid carrier incorporating nucleoside-modified tumor antigen encoding mRNA and the glycolipid antigen αGC. Vaccination with Galsomes activates two types of effector cells: CD8^+^ cytotoxic T lymphocytes (CTLs) and iNKT cells. To fully capitalize on these cells' ability to eradicate cancer cells, it is critical to understand which barriers within the tumor-bearing host might impede their activity. PD-L1 is such a barrier that can act on CTLs and iNKT cells both at the time of activation and at the time of their effector function. Here, we investigated the therapeutic effect of Galsomes as well as the dynamics of PD-L1 upon vaccination using SPECT/microCT imaging of a ^99m^Tc-radiolabeled anti-murine PD-L1 nanobody. Furthermore, two doses of the iNKT cell stimulating compound, αGC, were included in the study to assess its contribution to anti-tumoral responses as well as to PD-L1 expression.

In accordance with a previous study, we observed an anti-tumor immune response upon mRNA Galsome vaccination, as evidenced by an increase in iNKT cells and tumor-specific CD8^+^ T cells in systemic organs and tumor 11. The increase in iNKT cells correlated with the dose, which was expected as the presentation of αGC results in the activation and proliferation of iNKT cells, as previously shown *ex vivo* in peripheral blood cells 34. Although activation of iNKT cells results in a burst release of immune-stimulating cytokines and CD40/CD40L cross-talk with DCs, we did not detect elevated numbers of OVA-specific CD8^+^ T cells in the spleen using increasing αGC-doses 35. Fujii *et al*. previously evaluated the upregulation of maturation markers on DCs upon administration of increasing αGC-doses and observed that a strong shift in expression occurred at the lowest dose whereas higher doses only marginally increased the expression of maturation markers 36. Besides immune activation, we also evaluated the therapeutic potential of mRNA Galsomes containing increasing αGC-doses. We demonstrated that mRNA Galsomes attenuated tumor development and significantly increased the time to reach a humane endpoint in an αGC-dose-dependent manner. It should be noted that B16-OVA is a CD1d-negative tumor model, hence the malignant cells could not directly have been killed by iNKT cells 36. Although we did not observe a dose-dependent increase of OVA-specific CD8^+^ T cells in the spleen, lung and liver, analysis of tumor tissue revealed a dose-dependent increase of CTLs starting 10 days after vaccination, potentially explaining the therapeutic benefit of including higher αGC-doses.

A ^99m^Tc-labeled anti-PD-L1 nanobody was employed for noninvasive and repeated visualization of PD-L1 expression in mice. SPECT/CT images showed a strong and systemic upregulation of PD-L1 as early as 1 day after vaccination. As ligation of PD-L1 to PD-1 interferes with TCR signaling and attenuates the effector function of anti-tumoral T cells 18,37, these results provide a rationale for early PD-L1 blockade in combination with cancer vaccination. PD-L1 imaging also revealed that mice treated with a higher αGC-dose did not show higher expression of PD-L1 compared to mice treated with a lower dose. We hypothesize that, despite the higher cytokine secretion associated with higher doses of αGC, a threshold in PD-L1 expression was reached using the low αGC-dose. Furthermore, aGC resulted in iNKT activation in places where Galsomes accumulated as opposed to low fLuc mRNA expression outside of the lungs. Hence we compared PD-L1 upregulation and DiR based distribution of Galsomes. Interestingly, PD-L1 expression did not correlate to the prevalence of cytokine-secreting effector cells (iNKT and OVA-specific CD8^+^ T cells) as we observed that these cell types were only peaking later in the experiment. Although this finding suggests that PD-L1 expression is not correlated to the activity of these cells, it should be noted that iNKT cells secrete cytokines (*e.g.,* IFN-γ) as early as a few h after stimulation, long before their proliferation is detectable 38. Consequently, the rapid upregulation of PD-L1 could be attributed to cytokine release by iNKT cells. Additionally, even though nucleoside-modified mRNA was used, immunogenic effects of mRNA and/or the lipid carrier could also have induced cytokine release by cells that phagocytosed the mRNA lipoplexes. Indeed, it was shown that vaccination with lipid nanoparticles (LNPs) containing N1 methyl pseudo-uridine-modified mRNA results in a rapid secretion of cytokines (*e.g.,* IFN-γ, IFN-α and IL-6) 39. It has not been elucidated how mRNA vaccines trigger the innate immune system, both mRNA and the lipid carrier could possibly be involved 40. In this context, it is important to consider that here, mRNA was modified with m1Ѱ but not purified and consequently could have contained components (*e.g.*, dsRNA) that trigger certain pattern recognition receptors. To add on this, also the DOTAP-based cationic lipid nanocarrier that was used has the potential to stimulate innate immune sensors 41.

We observed that both lungs and liver, organs where uptake of Galsomes was shown, displayed a significantly higher uptake of ^99m^Tc-labeled anti-PD-L1 nanobodies, in comparison with untreated mice. Unfortunately, due to overlapping signals from liver and kidney, we were not able to visualize PD-L1 expression in the spleen, although this organ was also targeted by Galsomes. Interestingly, the data indicated that PD-L1 in the lungs is mainly elevated in non-immune cells upon vaccination. Cheng et al. reported that positively charged LNPs are targeted to the lungs and are massively taken up by non-immune cells (endothelial (66%) and epithelial (39%) cells), which possibly correlates with our observations that mainly non-immune cells contribute to PD-L1 expression in the lungs 31. However, it should be noted that PD-L1 expression is typically induced by secreted cytokines which could also act on more distant cells as well. In liver, vaccination-induced PD-L1 expression was short-lived, whereas in lungs the signal persisted and only gradually receded over time. Possibly, as we observed a higher prevalence of OVA-specific CD8^+^ T cells in the lungs compared to the liver, the prolonged signal observed in the lungs could be the result of the activation of T cells and thus prolonged secretion of cytokines. In addition, we showed that liver CD8^+^ and CD4^+^ T cells also upregulated PD-L1 expression only one day after vaccination. As PD-L1 is upregulated in stimulated T cells, we hypothesize that activation of T cells proceeded differently in both organs, possibly as a consequence of the variation in the cell types that internalize Galsomes in both organs 42 Furthermore, we observed that there was a pronounced infiltration of myeloid cells in the liver after vaccination. As we also observed systemic upregulation of PD-L1 shortly after vaccination, it is possible that PD-L1 expression observed in the liver could partially be attributed to infiltrating PD-L1-expressing cells. Indeed, Loacker *et al*. recently reported elevated PD-L1 expression in blood granulocytes and monocytes after COVID mRNA vaccination 43. In this light, it should also be noted that organs were not perfused before flow cytometry analysis, which could also influence the results as no discernment can be made between the activity in circulating blood and/or tissue upon imaging.

The tumor PD-L1-signal was particularly obvious in tumors that are 'small', *i.e.,* which are kept in control by the immune system 44. In tumors, radiotracer uptake is related to the vaccination showing some level of upregulation in mice treated with a high dose of αGC. Yet, there is no marked increase or decrease in time and appears to remain stable within the observation span of this experiment. This study shows that nanobody-mediated imaging of PD-L1 is beneficial and realistic for providing a rationale to not only act on PD-1/PD-L1 interactions during the effector phase but also during the priming phase. These results demonstrate the importance of the spatiotemporal evaluation of PD-L1 expression upon immune intervention (*e.g.,* vaccination) but could also be relevant for other treatment approaches. This study however argues to be cautious when interpreting images. Results obtained in lung and liver were confirmed by *ex vivo* analysis. Yet, this is not the case for tumor. Initial high PD-L1 expression in the periphery might act as a sink, capturing the nanobody radiotracer before it can penetrate the tumor. This argument is strengthened by the tracer activity found in bladder, which acts as a reservoir of radiolabeled nanobodies upon clearance via the urinary tract. In general, the amount of activity is much lower in the bladder of vaccinated mice compared to untreated mice at early timepoints, suggesting more tracer remains in either the blood circulation or peripheral tissue. Nonetheless, high resolution signals obtained with the nanobody-based PD-L1 radiotracer could allow a trained physician to correctly interpret the images, thereby opening perspectives to evaluate response to therapy based on high peripheral and tumor PD-L1 signals early after vaccination, and to propose an optimal combination regimen. To facilitate clinical translation, development of a PET anti-PD-L1 radiotracer would be warranted due to higher clinical value than SPECT tracers as this allows easier quantification of signals and comes with a higher spatial resolution and higher sensitivity.

In conclusion, we present a method to visualize PD-L1 expression upon mRNA vaccination in melanoma-tumor bearing mice via SPECT/CT imaging using a ^99m^Tc-labeled anti-PD-L1 nanobody. We showed that PD-L1 is rapidly and systemically upregulated upon vaccination with mRNA Galsomes and that mainly lung and liver were showing strong expression of this immune checkpoint. In these organs, both myeloid and non-immune cells showed higher expression of PD-L1 after vaccination. We also showed increased PD-L1 expression in tumor tissue which was mostly higher in mice with small tumors. To our knowledge, this is the first study that visualizes PD-L1 expression after mRNA vaccination and as such provides a rationale for combination therapy.

## Supplementary Material

Supplementary figures and tables.
