# Supplementary material for: Nanobody-mediated SPECT/CT imaging reveals the spatiotemporal expression of programmed death-ligand 1 in response to a CD8+ T cell and iNKT cell activating mRNA vaccine
Source: Theranostics. 2023 Oct 9;13(15):5483–500. doi: 10.7150/thno.85106 (PMC10614673; doi:10.7150/thno.85106)
Supplement: Supplementary file 1 — Supplementary figures and tables. [file thnov13p5483s1.pdf]

SUPPLEMENTARY DATA

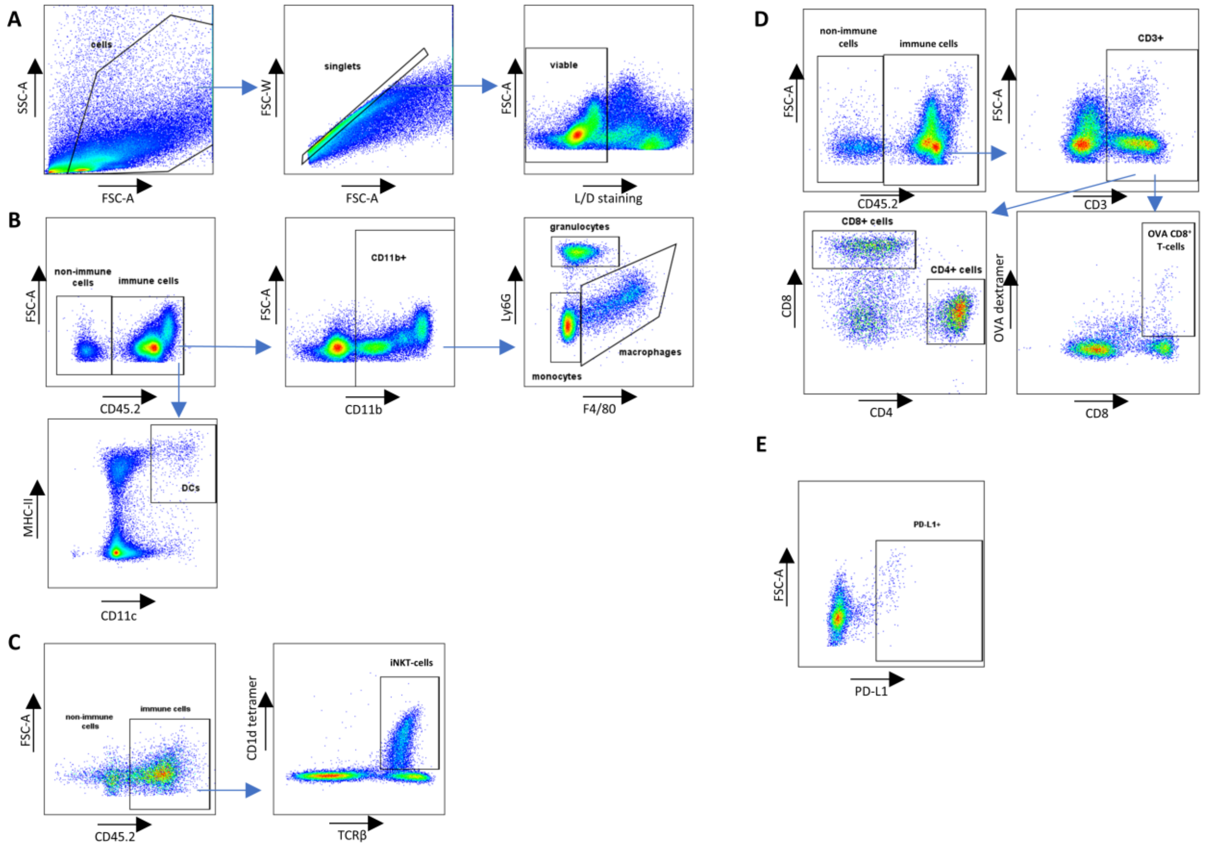

Figure S1: Flow cytometry gating strategy. Overview of the performed gating strategy. **(A)** Gating strategy for viable cells was performed for all flow cytometry panels. Viable cells were selected by gating all cells, single cells and viable cells. **(B)** Myeloid panel. Myeloid cells were selected by gating immune cells and CD11b^{+}-cells and were subdivided into different populations: granulocytes (F4/80^{lo}Ly6G^{hi}), macrophages (F4/80^{hi}) and monocytes (F4/80^{lo}Ly6G^{lo}). Dendritic cells (DCs) were selected via gating on CD11c^{hi} and MHCII^{hi} cells in the immune cell population. **(C)** Lymphoid panel. T lymphocytes were selected by gating immune cells and CD3^{+}-cells and were subdivided into CD4^{+} and CD8^{+} T cells. OVA-specific CD8^{+} T cells were selected via gating on OVA tetramer^{hi} and CD8^{hi} cells in CD3^{+}-cell population. **(D)** iNKT panel. iNKT cells were selected by gating CD1d^{hi} and TCRβ^{hi} cells in immune cell population **(E)** PD-L1 expression was defined for each individual cell type.

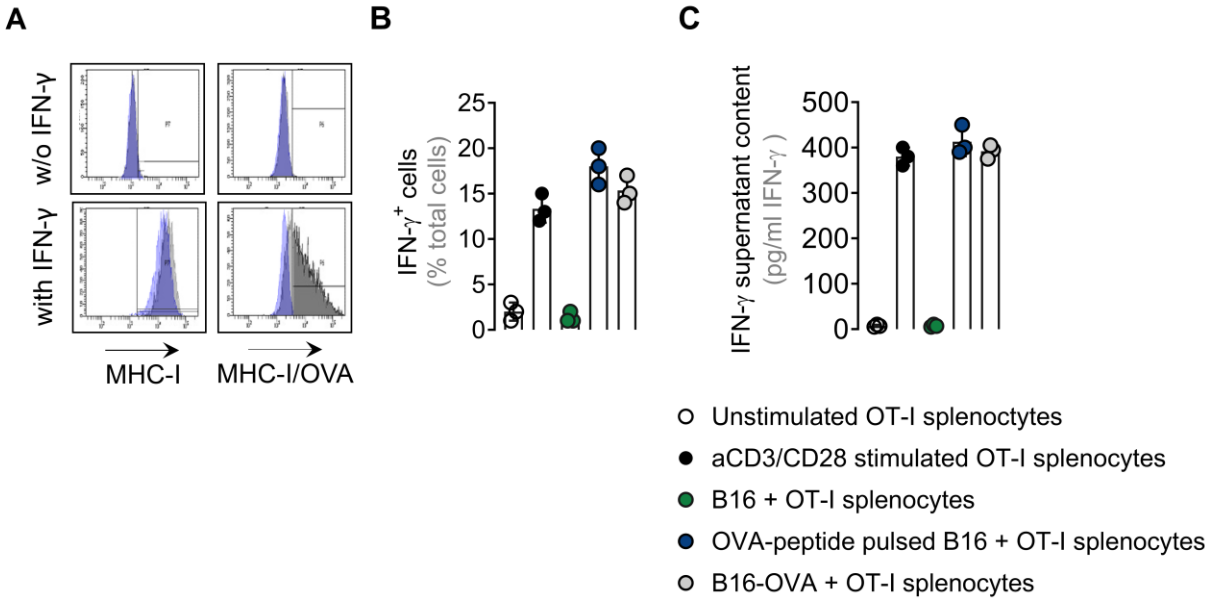

Figure S2: B16-OVA cells present OVA in MHC-I and are recognized by OVA-specific CD8$^+$ T cells. (A) Representative flow cytometry histograms showing expression of MHC-I or MHC-I/OVA complexes on the surface of B16 (blue), or B16-OVA (grey) cells cultured with or w/o IFN-γ (n=3). **(B,C)** OVA-specific CD8$^+$ T cells were isolated from the spleen of OT-I mice and co-cultured with B16 cells, B16 cells pulsed with the MHC-I restricted peptide of OVA or B16-OVA cells for 24 h. As technical controls, OT-I cells were cultured w/o further stimulation or were stimulated with aCD3/CD28 antibody-coated beads. **(B)** Flow cytometry was performed to evaluate the percentage of cells that produce IFN-γ upon coculture of OT-I splenocytes and B16 variants (n=3). **(C)** ELISA was performed to evaluate the amount of IFN-γ in the supernatants upon coculture of OT-I splenocytes and B16 variants (n=3). The bar graphs in **(B,C)** summarize the results as mean ± SD. Symbols represent individual data points.

A

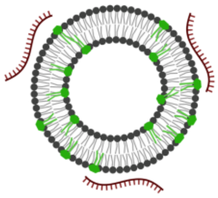

B

Galsome	Size ± SD (nm)	Polydispersity Index ± SD	Zeta Potential ± SD (mV)
Low αGC dose (2 ng)	275 ± 16.6	0.193 ± 0.01	46.8 ± 1
High αGC dose (20 ng)	267 ± 39.1	0.355 ± 0.02	46.1 ± 4

Figure S3: Galsomes containing a low or high αGC-dose show similar physicochemical properties. (A) Schematic representation of Galsomes. Lipoplexes composed of DOTAP, cholesterol and incorporating αGC and mRNA **(B)** Size (intensity mean), polydispersity index, and zeta-potential of Galsomes dispersed in HEPES buffer, formulated with 2 ng (low dose) or 20 ng (high dose) αGC (*i.e.*, 0.015 and 0.0015 mol% of total lipids respectively, n=3).

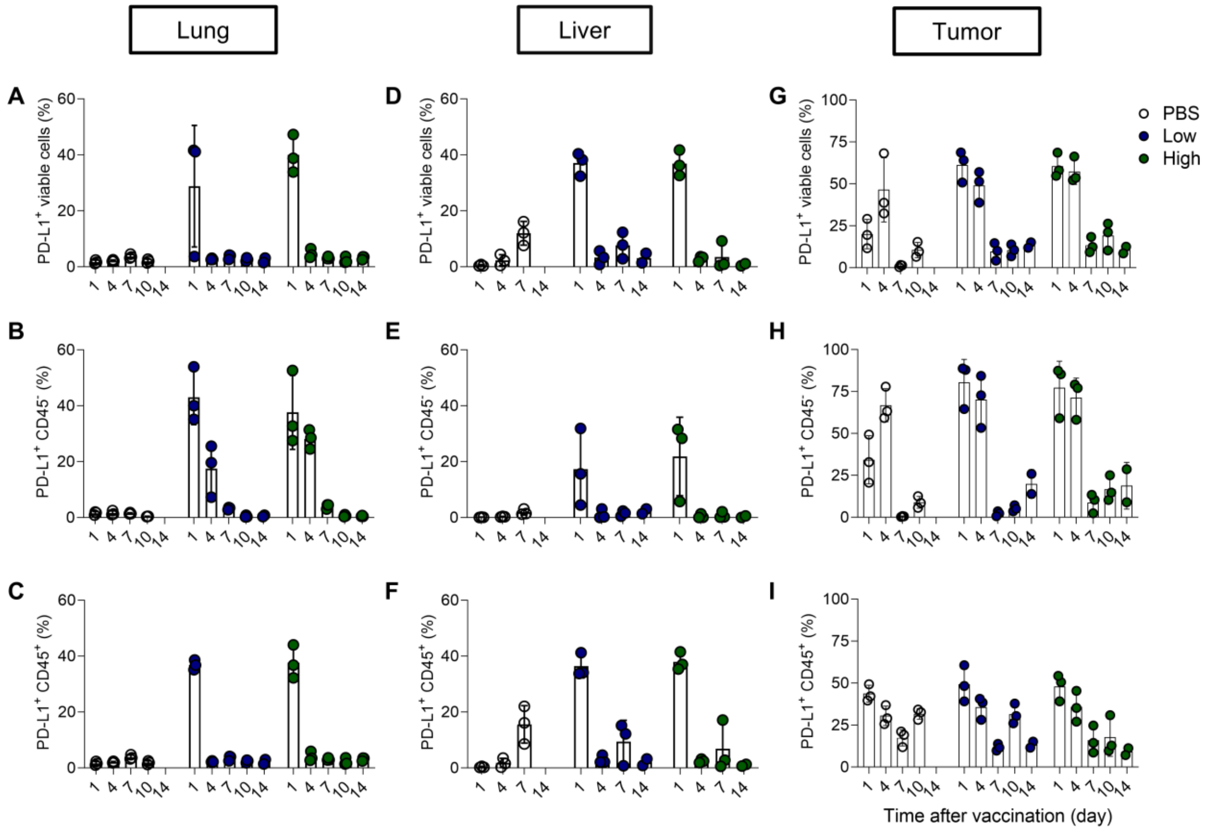

Figure S4: PD-L1 expression in lung, liver and tumor assessed by measuring PD-L1$^+$ cells shows the same trends as observed for PD-L1 MFI values. (A, D, G) Flow cytometry results represented as PD-L1$^+$ cells in the viable cell population of (A) lung, (D) liver and (G) tumor. **(B, E, H)** Flow cytometry results represented as PD-L1$^+$ cells in the non-immune cell population of (B) lung, (E) liver and (H) tumor. **(C, F, I)** Flow cytometry results represented as PD-L1$^+$ cells in the immune cell population of (C) lung, (F) liver and (I) tumor. All data are shown as mean ± SD (n=1, mpc=3).

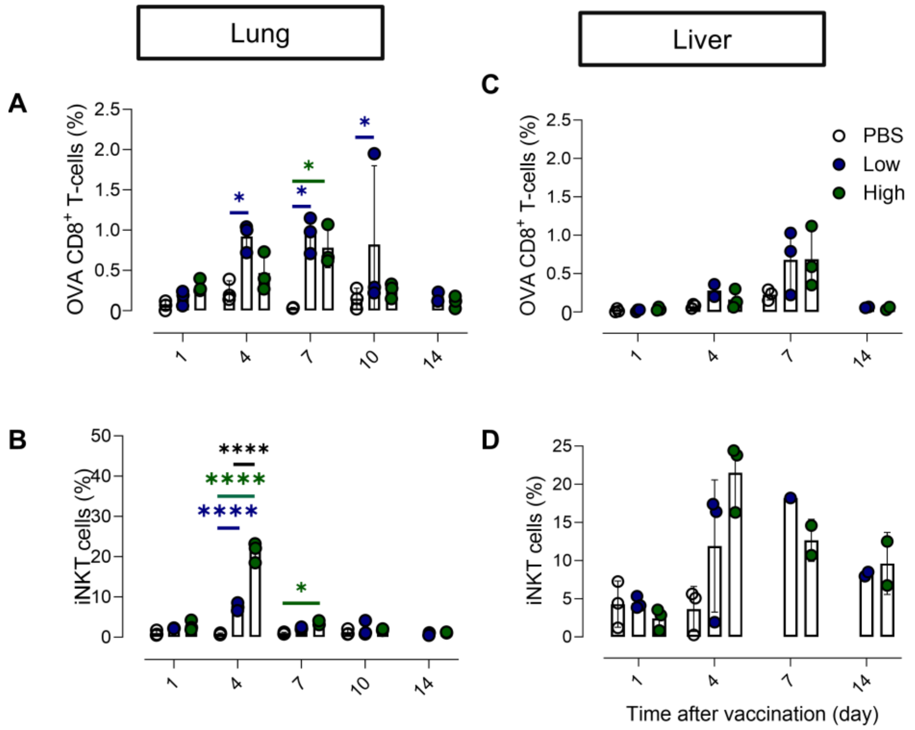

Figure S5: Proliferation of OVA-specific CD8$^+$ T cells and iNKT cells in lung and liver of treated and untreated mice. Graphs showing percentage of OVA-specific CD8+ T cells **(A)** and iNKT cells **(B)** within the CD45$^+$ population in the lung detected on days 1, 4, 7, 10 and 14 as mean ± SD. Statistical analysis was performed by two-way ANOVA followed by Tukey's *post hoc* test. (n=1, mpc=3).

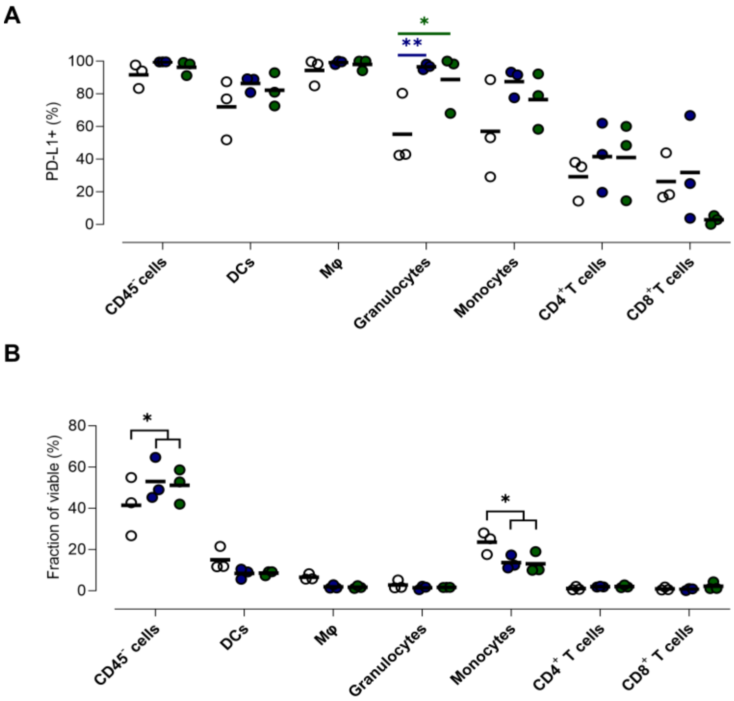

Figure S6: PD-L1 expression at cellular level in tumor 1 day after vaccination. (A) Fraction of PD-L1+ non-immune ($CD45^-$) cells, DCs, macrophages (Mφ), granulocytes, monocytes, $CD4^+$ and $CD8^+$ T cells within viable cells 1 day after vaccination with Galsomes containing a low (blue) or high (green) αGC-dose or PBS (white) in tumor. **(B)** Fraction of non-immune cells and different immune cell types within viable cells 1 day after vaccination. Symbols represent individual data points and mean is indicated by a line. Statistical analysis was performed by two-way ANOVA followed by Tukey's *post hoc* test. (n=1, mpc=3).

$PD\text{-}L1^{Tr}_{Lung} = \beta_0 + \beta_1 t + \alpha_1 T_{Low} + \alpha_2 T_{High} + \gamma_{11} t T_{Low} + \gamma_{12} t T_{High}$			
**Predictors**	Estimates	confidence interval	p-value
β_0	1.15	1.06 – 1.24	<0.001
β_1	-0.01	-0.03 – 0.00	0.062
α_1	-0.83	-0.94 – -0.72	<0.001
α_2	-0.83	-0.94 – -0.73	<0.001
γ_{11}	0.05	0.03 – 0.07	<0.001
γ_{12}	0.06	0.04 – 0.07	<0.001
**Observations**	61		
**R^2**	0.916		
**AIC**	-122.953		

Table S1: Model Summary of $PD\text{-}L1^{Tr}_{Lung}$ based on imaging data.

**Contrast**	**Estimate**	**SE**	**df**	**t.ratio**	**p-value**
**PBS - Low**	-0.05031	0.00802	55	-6.269	<0.0001 (difference)
**PBS - High**	-0.05789	0.00792	55	-7.312	<0.0001 (difference)
**Low - High**	-0.00759	0.00534	55	-1.422	0.3369 (no difference)

Table S2: Pairwise comparison of PD-L1 signal in lungs between treatment conditions adjusted with TUKEY. SE = Standard error; df = degrees of freedom.

$$PD\text{-}L1^{Tr}_{Liver} = \beta_0 + \beta_1 t + \alpha_1 T_{Low} + \alpha_2 T_{High} + \gamma_{11} t T_{Low} + \gamma_{21} t^2 T_{Low} + \gamma_{12} t T_{High} + \gamma_{22} t^2 T_{High}$$

**Predictors**	Estimates	confidence interval	p-value
β_0	0.449	0.39 – 0.51	<0.001
β_1	-0.016	-0.04 – 0.01	0.209
β_2	0.001	-0.00 – 0.00	0.253
α_1	-0.267	-0.34 – -0.19	<0.001
α_2	-0.235	-0.31 – -0.16	<0.001
γ_{11}	0.061	0.03 – 0.09	<0.001
γ_{21}	-0.003	-0.01 – -0.00	0.006
γ_{12}	0.054	0.02 – 0.08	0.001
γ_{22}	-0.003	-0.01 – -0.00	0.017
**Observations**	61		
**R^2**	0.751		
**AIC**	-211.925		

Table S3: Model Summary of $PD\text{-}L1^{Tr}_{Liver}$ based on imaging data.

**Contrast**	**Estimate**	**SE**	**df**	**t.ratio**	**p-value**
**PBS - Low**	-0.013837	0.00472	52	-2.930	0.0137 (difference)
**PBS - High**	-0.013334	0.00469	52	-2.844	0.0172 (difference)
**Low - High**	0.000502	0.00272	52	0.185	0.9813 (no difference)

Table S4: Pairwise comparison of PD-L1 signal in liver between treatment conditions adjusted with TUKEY.

$\boldsymbol{PD\text{-}L1^{Tr}_{Tumor} = \beta_0 + \beta_1 t + \alpha_1 T_{Low} + \alpha_2 T_{High}}$			
**Predictors**	Estimates	confidence interval	p-value
$\boldsymbol{\beta_0}$	0.463	0.357 – 0.568	<0.001
$\boldsymbol{\beta_1}$	0.002	-0.002 – 0.006	0.384
$\boldsymbol{\alpha_1}$	0.009	-0.150 – 0.167	0.907
$\boldsymbol{\alpha_2}$	-0.140	-0.288 – 0.007	0.061
$\boldsymbol{\sigma^2}$	0.011		
$\boldsymbol{\tau_{00\ Mouse}}$	0.008		
**ICC**	0.421		
**N $_{Mouse}$**	14		
**Observations**	61		
**Marginal R^2 / Conditional R^2**	0.208 / 0.541		
**AIC**	-70.969		

Table S5: Mixed model summary of $PD\text{-}L1^{Tr}_{Tumor}$ based on imaging data. Contrary to the data in liver and lung the mixed model in tumor present better fitting than an only fixed effect model.

Contrast	Estimate	SE	df	t.ratio	p-value	
**PBS - Low**	-0.00858	0.0721	11	-0.119	0.9922	(No difference)
**PBS - High**	0.14013	0.0671	11	2.090	0.1374	(No difference)
**Low - High**	0.14871	0.0678	11	2.194	0.1163	(No difference)

Table S6: Pairwise comparison of PD-L1 signal in tumor tissue between treatment conditions adjusted with TUKEY.
